# Supplementary material for: Proteomic Analysis of Mesenchymal Stromal Cell-Derived Extracellular Vesicles and Reconstructed Membrane Particles
Source: Int J Mol Sci. 2021 Nov 29;22(23):12935. doi: 10.3390/ijms222312935 (PMC8657583; doi:10.3390/ijms222312935)
Supplement: Supplementary file 1 [file ijms-22-12935-s001.zip › ijms-1483891-Supplementary.pdf]

Supplementary Materials

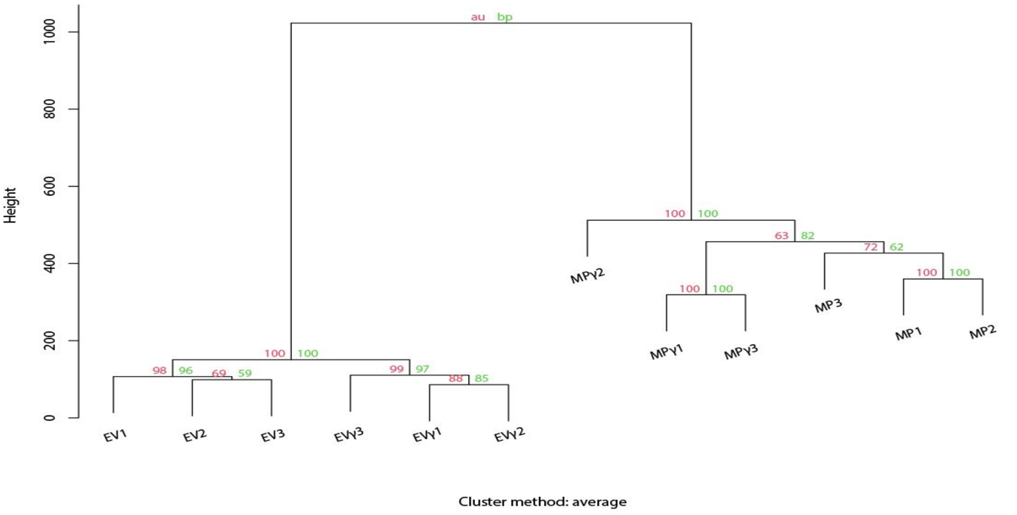

Supplementary Figure S1. Hierarchical clustering of EV, EVγ, MP and MPγ. P-values were computed for each of the clusters found via multiscale bootstrap resampling. Values at branches are p-values (left) and bootstrap probability values (right).

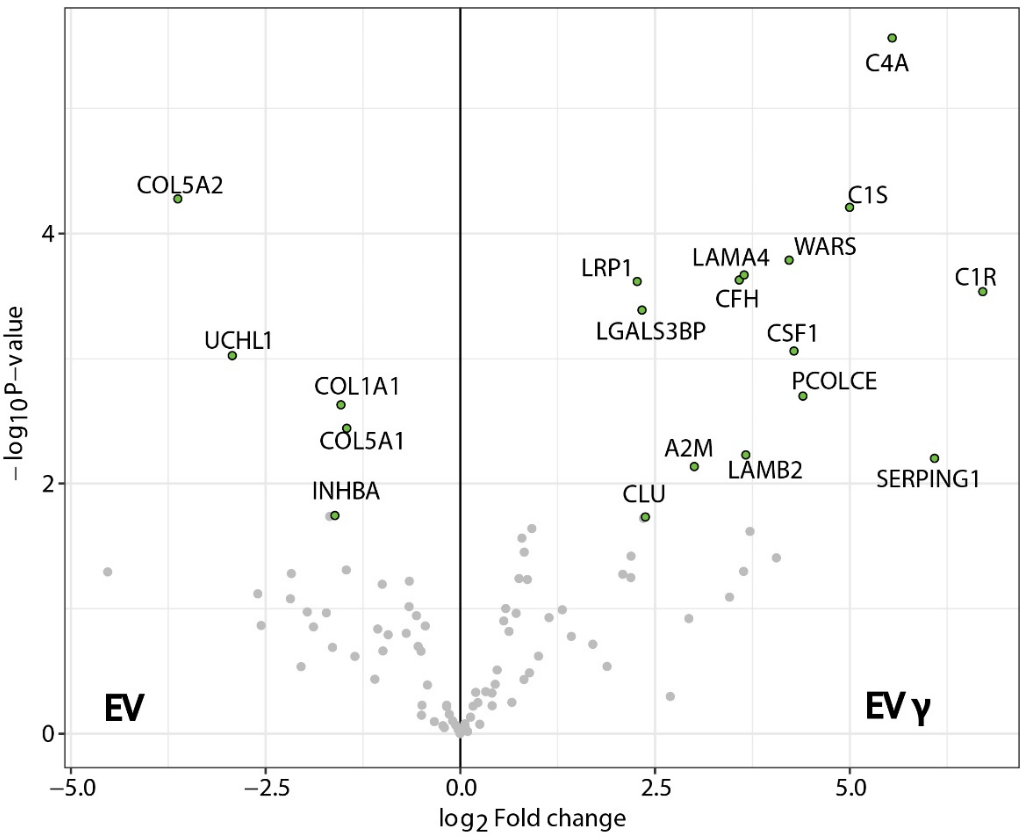

Supplementary Figure S2. Volcano plot showing down and upregulated genes in EV vs EV.

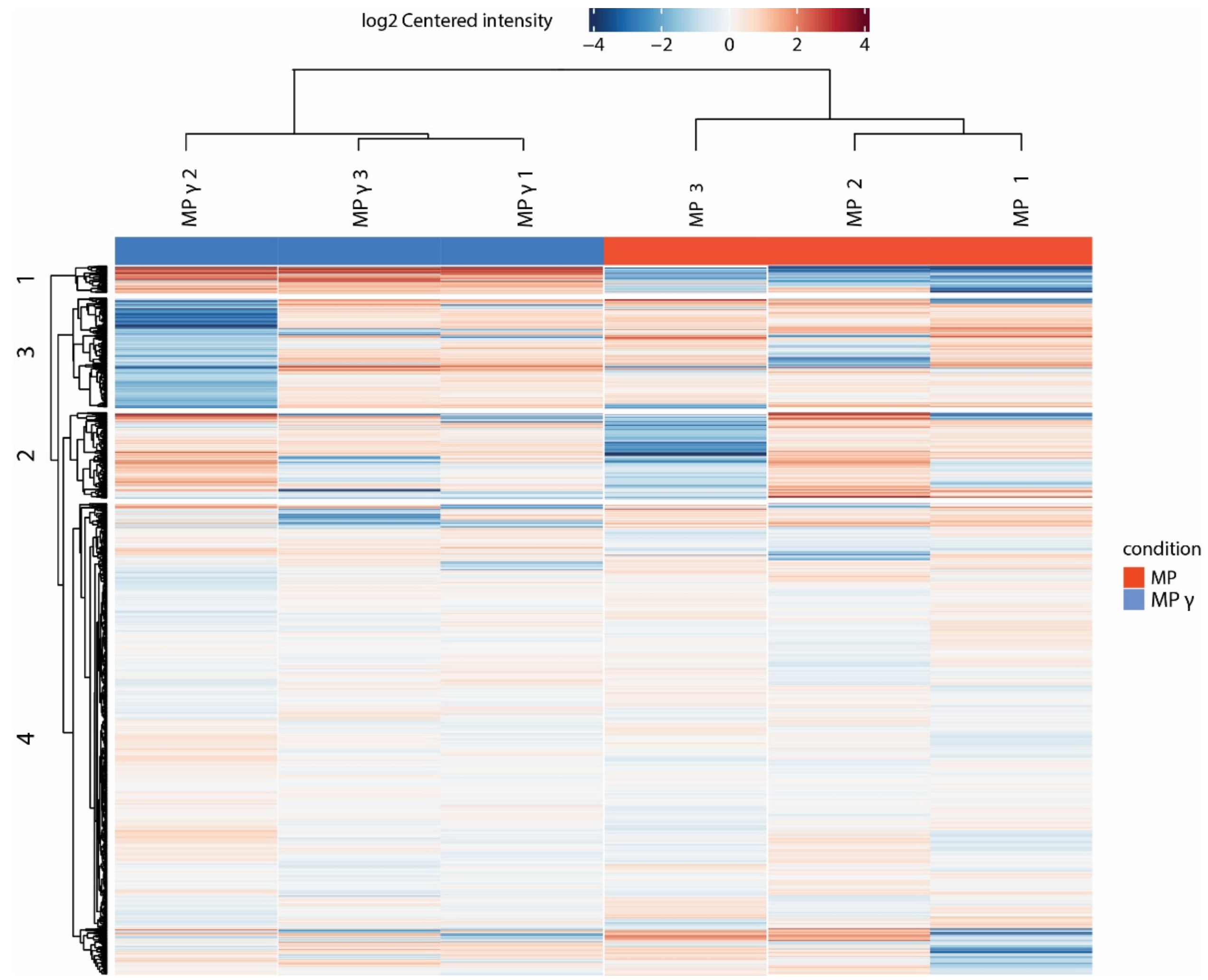

Supplementary Figure S3. Heatmap with all significant enriched proteins in all MP samples (p-value ≤ 0.05). Hierarchical clustering using average linkage shows that IFNγ has a strong effect on the protein composition of MP.

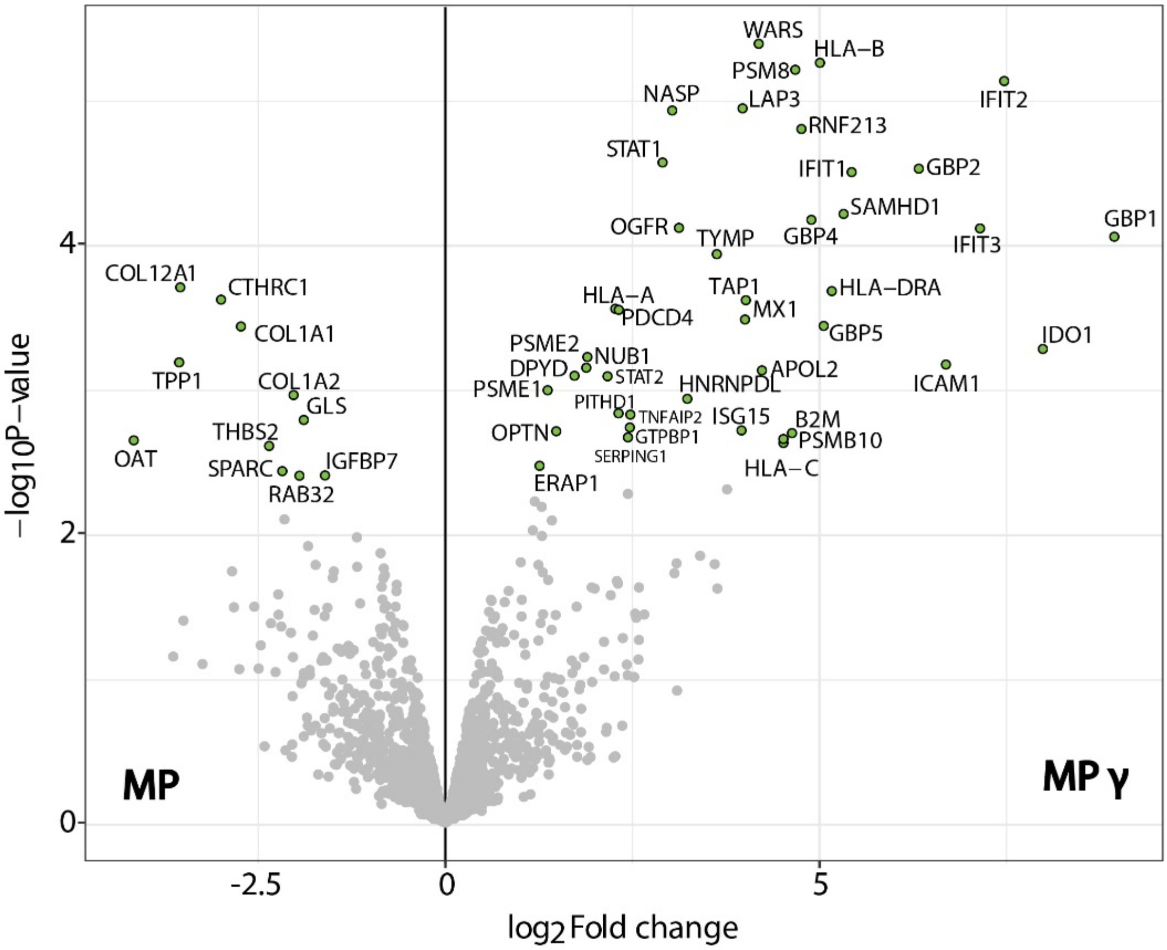

Supplementary Figure S4. Volcano plot showing down and upregulated genes in MPγ vs MP.

Supplementary Table S1. List of proteins shown in the Venn diagram of Figure 3A, indicating overlapping expression in EV, EVγ, MP and MPγ.

Pathway ID	Description	Fold enrichment	P value	Upregulated genes	Downregulated genes
R-HSA-1474244	Extracellular matrix organization	23.967644	3.5e-12	A2M, DCN, EFEMP1, FBLN5, FN1, HTRA1, LAMA4, LAMB2, LAMC1, MMP2, PCOLCE, PLOD1, TIMP2	COL12A1, COL1A1, COL1A2, COL3A1, COL4A1, COL5A1, COL5A2, COMP, FMOD, HAPLN1, HSPG2, LOXL2, LUM, SERPINE1, SERPINH1, SPARC, THBS1, TIMP1, VCAN

R-HSA-2173782	Binding and Uptake of Ligands by Scavenger Receptors	23.339460	1.6e-10	LRP1	COL1A1, COL1A2, COL3A1, COL4A1, SPARC, SSC5D
R-HSA-8948216	Collagen chain trimerization	35.293817	4.3e-10		COL12A1, COL1A1, COL1A2, COL3A1, COL4A1, COL5A1, COL5A2
R-HSA-2022090	Assembly of collagen fibrils and other multimeric structures	30.625323	1.8e-09	PCOLCE	COL1A1, COL1A2, COL3A1, COL4A1, COL5A1, COL5A2, LOXL2
R-HSA-75035	Chk1/Chk2(Cds1) mediated inactivation of Cyclin B:Cdk1 complex	47.704830	3.6e-09		YWHAB, YWHAE, YWHAZ
R-HSA-1650814	Collagen biosynthesis and modifying enzymes	33.342086	3.7e-09	PCOLCE, PLOD1	COL12A1, COL1A1, COL1A2, COL3A1, COL4A1, COL5A1, COL5A2, SERPINH1
R-HSA-111447	Activation of BAD and translocation to mitochondria	41.344186	6.9e-09		YWHAB, YWHAE, YWHAZ
R-HSA-114608	Platelet degranulation	28.571185	1.2e-08	A2M, CLU, FN1, LGALS3BP, PSAP, QSOX1, SERPING1, TLN1, VCL	CALU, ECM1, FLNA, ISLR, SERPINE1, SPARC, THBS1, TIMP1
R-HSA-76005	Response to elevated platelet cytosolic Ca2+	27.455123	1.6e-08	A2M, CLU, FN1, LGALS3BP, PSAP, QSOX1, SERPING1, TLN1, VCL	CALU, ECM1, FLNA, ISLR, SERPINE1, SPARC, THBS1, TIMP1
R-HSA-1474290	Collagen formation	27.070598	1.8e-08	PCOLCE, PLOD1	COL12A1, COL1A1, COL1A2, COL3A1, COL4A1, COL5A1, COL5A2, LOXL2, SERPINH1

Supplementary Table S2. Display of enriched pathways in EVγ compared to EV, detailing the fold enrichment, p-value and all up and down regulated genes in the pathways.

Pathway ID	Description	Fold enrichment	P value	Upregulated genes	Downregulated genes
R-HSA-379716	Cytosolic tRNA aminoacylation	15.1173469	8.4e-20	CARS, EEF1E1, KARS, PPA1, WARS	AARS, AIMP1, AIMP2, DARS, EPRS, IARS, LARS, MARS, NARS, QARS, RARS, TARS, VARS
R-HSA-379724	tRNA Aminoacylation	9.1183997	1.1e-16	CARS, EEF1E1, KARS, PPA1, WARS	AARS, AIMP1, AIMP2, DARS, EPRS, IARS, IARS2, LARS, MARS, NARS, QARS, RARS, TARS, VARS
R-HSA-913531	Interferon Signaling	3.8880486	9.8e-13	B2M, CAMK2D, DDX58, EIF2AK2, EIF4E, GBP1, GBP2, GBP5, HLA-DRA, HLA-DRB1, ICAM1, IFI35, IFIT1, IFIT2, IFIT3, IFITM1, ISG15, MAPK3, MX1, NEDD4, PML, SAMHD1, SP100, STAT1, STAT2, TPR, TRIM21, TRIM25, TRIM38, UBA7, UBE2L6	ABCE1, EIF4A2, EIF4G2, EIF4G3, FLNA, KPNA4, RANBP2
R-HSA-1169408	ISG15 antiviral mechanism	5.0391156	1.5e-12	DDX58, EIF2AK2, EIF4E, IFIT1, ISG15, MAPK3, MX1, NEDD4, STAT1, TPR, TRIM25, UBA7, UBE2L6	EIF4A2, EIF4G2, EIF4G3, KPNA4, RANBP2
R-HSA-1169410	Antiviral mechanism by IFN-stimulated genes	5.0391156	4.5e-12	DDX58, EIF2AK2, EIF4E, IFIT1, ISG15, MAPK3, MX1, NEDD4, STAT1, TPR, TRIM25, UBA7, UBE2L6	ABCE1, EIF4A2, EIF4G2, EIF4G3, FLNA, KPNA4, RANBP2

R-HSA-72203	Processing of Capped Intron-Containing Pre-mRNA	1.9316610	3.8e-11	EIF4E, ELAVL1, HNRNPH1, HNRNPR, HNRNPU, HNRNPUL1, PRPF19, PTBP1, SNRPG, SRSF2, SRSF7, TPR	HNRNPA1, HNRNPA3, HNRNPD, HNRNPK, LSM2, POLR2H, RANBP2, SNRNP70, SNRPF, SRSF3, SRSF4
R-HSA-8856825	Cargo recognition for clathrin-mediated endocytosis	4.9403095	9.0e-11	AP2A2, COPS3, COPS4, COPS7B, EPS15, SCARB2, STAM2, TGOLN2, UBQLN1	AP2A1, AP2M1, CLTB, COPS5, COPS8, DAB2, EPN1, EPS15L1, FCHO2, GPS1, HGS, NEDD8, PICALM, SH3GL1, STAM, TFRC
R-HSA-917937	Iron uptake and transport	3.8898437	1.6e-10	ACO1, ALAD, ATP6V1A, ATP6V1B2, ATP6V1C1, CUL1, FTH1	NEDD8, SKP1, TCIRG1, TFRC
R-HSA-72163	mRNA Splicing - Major Pathway	2.2647711	1.8e-10	ELAVL1, HNRNPH1, HNRNPR, HNRNPU, HNRNPUL1, PRPF19, PTBP1, SNRPG, SRSF2, SRSF7	HNRNPA1, HNRNPA3, HNRNPD, HNRNPK, LSM2, POLR2H, SNRNP70, SNRPF, SRSF3, SRSF4
R-HSA-8951664	Neddylation	1.67237039	1.8e-10	COMMD1, COPS3, COPS4, COPS7B, CUL1, CUL2, CUL3, NUB1, UBA3	COMMD4, COPS5, COPS8, DCUN1D1, DDB1, GPS1, LMO7, NEDD8, RBBP7, SKP1
